# Serum complement 3 is a potential biomarker for assessing disease activity in Takayasu arteritis

**DOI:** 10.1186/s13075-021-02433-x

**Published:** 2021-02-24

**Authors:** Rongyi Chen, Lingying Ma, Peng Lv, Jiang Lin, Chaolun Li, Yan Yan, Xuejuan Jin, Xiaomin Dai, Zongfei Ji, Huiyong Chen, Lili Ma, Ying Sun, Lindi Jiang

**Affiliations:** 1 Department of Rheumatology, Zhongshan Hospital, Fudan University, Shanghai, China; 2 Evidence-Based Medicine Centre, Fudan University, No.180, Fenglin Road, Xuhui District, Shanghai, 200032 China; 3 Department of Radiology, Zhongshan Hospital, Fudan University, Shanghai, China; 4 Department of Ultrasound, Zhongshan Hospital, Fudan University, Shanghai, China; 5 Cardiovascular Epidemiology, Zhongshan Hospital, Fudan University, Shanghai, China

**Keywords:** Complement 3, Takayasu arteritis, Disease activity, Biomarker, C-reactive protein

## Abstract

**Background:**

Takayasu arteritis (TA) is a rare disease, lacking convenient and feasible biomarkers to identify disease activity. We aimed to evaluate the value of complements in distinguishing active TA.

**Methods:**

Consecutive patients were enrolled from the prospective East China TA cohort from April 2008 to June 2019. Patients were divided into two groups according to their baseline Kerr score. The value of complements and other biomarkers in identifying disease activity were analysed with cluster analysis, ROC curves, and combined tests. An independent group of patients from July 2019 to December 2019 were employed to validate the results.

**Results:**

Of the enrolled 519 patients, 406 (72.2%) cases were identified as active disease. Higher erythrocyte sedimentation rate (ESR), C-reactive protein (CRP), interleukin-6 (IL-6), and complement 3 (C3) levels were observed in the active group. Elevated C3 (≥ 1.085 g/L) had a high value to identify active TA with a sensitivity of 69.9%, specificity of 66.7%, and AUC of 0.715. Combining the CRP (≥ 10.65 g/L; sensitivity, 50.7%; specificity, 82.4%) and C3, the sensitivity could be improved to 85.1% in parallel test and the specificity could be improved to 94.1% in serial test. Validation was further performed to confirm the value of C3 for disease activity assessment. The accuracy of the parallel test of CRP and C3 in external validation with independent 53 TA cases was 72.73% with the AUC of 0.721.

**Conclusion:**

Elevated C3 could effectively evaluate the disease activity of TA, and C3 combining with CRP could further improve the disease activity evaluation.

**Supplementary Information:**

The online version contains supplementary material available at 10.1186/s13075-021-02433-x.

## Background

Takayasu arteritis (TA), a chronic non-specific inflammatory vascular disease with unknown aetiology, is accompanied by high lethality and morbidity rates [1, 2]. Acute and uncontrolled chronic vascular inflammation would cause vessel wall injury, leading to vascular fibrotic repair in the late phase, and finally resulting in vascular remodelling [2, 3]. Therefore, quick and effective treatment is essential to control disease activity and to delay or interrupt vascular inflammation as well as subsequent destruction.

Precise assessment of disease activity plays important roles in the selection and adjustment of treatment strategy. However, valuable biomarkers to evaluate disease activity timely and accurately are still lacking. Traditional activity biomarkers including erythrocyte sedimentation rate (ESR) and C-reactive protein (CRP) are easily affected by other factors such as infections, pregnancy and so on, resulting in poor specificity [4–6]. Previous studies have reported potential biomarkers including matrix metalloproteinases (MMPs) and pentraxin (PTX)-3 which were elevated in active TA; however, their values in distinguishing TA disease activity had not been fully illustrated yet, which limited their applications in clinical practice [7]. Thus, it is crucial to find new biomarkers with high sensitivity and specificity to identify disease status of TA.

Complements are important immune molecules in innate immunity and involved in the pathogenesis of vasculitis [8, 9]. Decreased complement 3 (C3) is a widely used biomarker for active disease in systemic lupus erythematosus (SLE) [10]. Additionally, the downstream cleaved protein of C3, known as C5a, has become a promising target for ANCA-associated vasculitis (AAV) treatment [11, 12]. However, the role of complement, especially C3, in evaluating disease activity of TA has not been investigated yet. So, the aim of this study was to evaluate the potential value of complements in identifying disease activity of Takayasu arteritis.

## Methods

### Study design

A prospectively ongoing observational cohort—the East China Takayasu arteritis (ECTA) cohort—was established since 2010 centred at Zhongshan Hospital, Fudan University, Shanghai, China. All the registered patients in ECTA cohort were diagnosed as TA by experts according to the American College of Rheumatology (ACR) 1990 classification criteria [13]. Patients’ information was collected with a standardized form and stored at the Redcap database once diagnosed.

In the current study, baseline information including clinical characteristics, biomarkers, and medications were used for analysis. Patients complicated with the following diseases were excluded: (i) malignant tumour; (ii) acute infections (e.g. tuberculosis, hepatitis); and (iii) other autoimmune diseases (e.g. SLE, AAV). The baseline data of patients enrolled from April 2008 to June 2019 was extracted as the original dataset, while an independent group of patients from July 2019 to Dec 2019 were obtained as the validation dataset. The flowchart of study is listed in the Supplementary Fig. S1.

The investigation protocol was conformed with the Helsinki Declaration and approved by the Ethics Committee of Zhongshan Hospital, Fudan University (Approval No.: B2016-168). All the patients signed the informed consents prior to the enrolment.

### Laboratory tests

Blood samples at baseline of each patient were obtained and examined in the clinical laboratory of the hospital with standard operating procedure. In detail, ESR was detected by Westergren method; CRP as well as globulin and immunoglobulin was detected by automatic biochemical analyser; and cytokines were detected by chemiluminescent immunoassay (CLIA) using Semen platform. Serum complements were detected by immunity transmission turbidity (ITA) in routine procedures according to the instructions of the manufactures with automatic biochemical analyser.

### Disease activity evaluation

Kerr criteria were used as the gold standard for disease activity evaluation: (i) systemic symptoms (infection, tumour, etc., were excluded); (ii) elevated ESR levels; (iii) vascular ischemic symptoms or signs (weakened pulse or pulselessness, vascular bruits, or asymmetric blood pressure); and (iv) positive imaging results. New onset or worsening of two or more criteria indicated “active disease” [14]. Whole body enhanced magnetic resonance angiography (MRA) was performed instead of the traditionally used angiography specified in the Kerr criteria in each patient. Imaging types were identified according to the angiographic classification of the international TA conference in Tokyo (1996) based on lesion distribution [15].

### Statistics

Continuous variables are expressed as the mean ± standard deviation or median (interquartile range, IQR) and compared using the *t* test or *Wilcoxon* rank-sum test where appropriate. Categorical variables are presented as number (frequency) and compared with the *chi*-square test. The Spearman correlation analysis was employed to validate the relationship between C3 and other biomarkers. The univariate logistic regression analysis was executed to unearth the factors associated with disease activity, which was further observed reversely by cluster analysis with K-means method and principal component analysis based on the optimal number of groups determined by scree plots. Items with *P* < 0.05 in univariate logistic regression analysis were enrolled in the multivariate logistic regression analysis and performed the receiver operating characteristic (ROC) curves. Youden Index was employed to determine the optimal cut-off value and the corresponding diagnosis ability. The parallel test and serial test were further performed to evaluate the value of C3 in combined tests. Next, net reclassification index (NRI) and integrated discrimination index (IDI) were calculated to confirm the value of C3 in improving the ability of CRP or ESR in distinguishing active TA. In addition, the identification effect of the parallel test (CRP and C3) was validated by 10-fold cross-validation and external validation with the original dataset and the independent dataset respectively. A *P* < 0.05 was considered to be significant with a two-side test. The data were analysed using SPSS 22.0 (Chicago, IL, USA) and R software (Murray Hill, NJ, USA). The graphs were generated with Prism GraphPad 8.0 (San Diego, CA, USA) and R software.

## Results

### Patient characteristics

In total, 519 patients were enrolled. Of these, 427 cases (82.3%) were female. The mean age was 32 (24–45) years, and the median disease course was 21 (4.0–70.5) months. The most common manifestation was dizziness (41.4%), followed by fatigue (33.3%) and chest pain (22.2%). Pulselessness was observed in 41.8% of patients, vascular bruit in 44.5% cases, and both were more commonly observed in the active group. The most common imaging type was type V (35.1%), followed by type I (28.5%), type IV (10.2%), type IIb (7.7%), type IIa (7.1%), and type III (3.9%) (Table 1).

**Table 1:** The characteristics of TA patients in different disease activity status

	Total (***n*** = 519)	Inactive (***n*** = 113)	Active (***n*** = 406)	***P*** value
Age, years	32.0 (24.0–45.0)	32.0 (23.0–44.0)	32.0 (24.0–46.0)	0.585
Female, *n* (%)	427 (82.3)	89 (78.8)	338 (83.3)	0.269
Course, months	21.0 (4.0–70.5)	20.5 (5.0–60.0)	23.0 (3.0–71.0)	0.909
**Symptoms**				
Fever, *n* (%)	71 (13.7)	12 (11.3)	59 (14.8)	0.366
Fatigue, *n* (%)	173 (33.3)	29 (25.7)	144 (35.5)	0.011*
Night sweat, *n* (%)	16 (3.1)	0 (0)	16 (4.0)	0.030*
Dizziness, *n* (%)	215 (41.4)	44 (41.9)	171 (43.0)	0.845
Neck pain, *n* (%)	40 (7.7)	6 (5.6)	34 (8.5)	0.316
Chest pain, *n* (%)	115 (22.2)	15 (14.2)	100 (24.9)	0.018*
Abdominal pain, *n* (%)	14 (2.7)	0 (0)	14 (3.5)	0.048*
**Signs**				
Pulseless, *n* (%)	217 (41.8)	33 (29.2)	184 (45.3)	0.034*
Vascular bruit, *n* (%)	231 (44.5)	24 (24.0)	207 (52.3)	< 0.001*
**ESR**, mm/H	33.0 (14.0–59.0)	15.0 (7.0–23.0)	40.0 (18.0–66.5)	< 0.001*
**Vascular stenosis**, *n* (%)	426 (82.1)	82 (72.6)	344 (84.7)	0.882
**Vascular thickening**, *n* (%)	280 (53.9)	40 (35.4)	240 (59.1)	0.001*
**Type**, *n* (%)				
I	148 (28.5)	32 (28.3)	116 (28.6)	
IIa	37 (7.1)	9 (8.0)	28 (6.9)	
IIb	40 (7.7)	2 (1.8)	38 (9.4)	
III	20 (3.9)	5 (4.4)	15 (3.7)	
IV	53 (10.2)	15 (13.2)	38 (9.4)	
V	182 (35.1)	31 (27.4)	151 (37.2)	0.063
Hypertension, *n* (%)	111 (21.4)	26 (23.0)	85 (20.9)	0.145
Hyperlipidemia, *n* (%)	13 (2.5)	4 (3.5)	9 (2.2)	0.261
Haemoglobin, g/L	118.5 (106.0–130.0)	124.0 (115.0–133.0)	117.0 (105.0–129.0)	< 0.001*
WBC, × 10^9^/L	7.5 (6.0–9.8)	7.3 (6.0–8.8)	7.5 (6.0–9.9)	0.325
Platelet, × 10^9^/L	265.0 (213.0–342.0)	250.0 (210.0–302.5)	275.0 (213.5–353.0)	0.048*
Albumin, g/L	40.0 (37.0–43.0)	42.0 (39.0–44.2)	39.0 (36.2–42.1)	< 0.001*
Globin, g/L	29.0 (26.0–34.0)	27.0 (24.0–33.0)	30.0 (26.0–35.0)	< 0.001*
Creatinine, μmol/L	58.0 (49.0–70.0)	60.0 (50.0–73.0)	57.5 (49.0–69.0)	0.564
**Immunity-related markers**				
CRP, mg/L	7.5 (1.9–30.6)	2.4 (0.8–8.0)	10.8 (2.4–39.1)	< 0.001*
TNF-α, pg/mL	7.7 (5.8–10.2)	7.2 (5.9–10.1)	7.9 (5.8–10.30	0.692
IL-6, pg/mL	5.3 (2.4–11.2)	3.7 (2.1–7.4)	5.7 (2.7–12.3)	0.010*
C3, g/L	1.15 (0.99–1.33)	1.00 (0.91–1.15)	1.19 (1.03–1.56)	< 0.001*
C4, g/L	0.24 (0.20–0.29)	0.22 (0.19–0.27)	0.25 (0.20–0.30)	0.040*
CH50, g/L	65.2 ± 18.2	59.3 ± 17.9	66.4 ± 18.1	0.015*
IgG, g/L	13.1 (10.5–16.3)	11.7 (9.4–13.8)	13.3 (10.8–6.6)	0.002*
IgA, g/L	2.6 (1.8–3.6)	2.3 (1.7–3.0)	2.6 (1.9–3.8)	0.013*
IgM, g/L	1.4 (1.1–2.0)	1.4 (1.0–1.8)	1.5 (1.1–2.0)	0.160
IgE, g/L	26.0 (13.0–82.3)	25.0 (12.0–70.0)	26.0 (13.0–84.5)	0.982
**Medication history**				
Prednisone, *n* (%)	78 (15.0)	15 (13.3)	63 (15.5)	0.502
Methotrexate, *n* (%)	11 (2.1)	0 (0)	11 (2.7)	0.134
Cyclophosphamide, *n* (%)	58 (11.2)	17 (15.0)	41 (10.1)	0.157

*ESR* erythrocyte sedimentation rate, *WBC* white blood cells, *CRP* C-reactive protein, *TNF-α* tumour necrosis factor-α, *IL-6* interleukin-6, *C3* complement 3, *C4* complement 4, *CH50* median hemolytic complement, *IgA* immunoglobin A, *IgG* immunoglobin G, *IgM* immunoglobin M, *IgE* immunoglobin E

*P < 0.05

### Comparisons of disease characteristics between active and inactive patients

According to Kerr criteria, 406 cases (72.2%) were defined as active disease. There was no significant difference between active and inactive groups in age, sex, disease course, and imaging types. Patients with active disease had higher frequency of fatigue, night sweat, chest pain, abdominal pain, pulselessness, and vascular bruit than patients with inactive disease (*P* < 0.05). The prednisone, cyclophosphamide, and methotrexate were the most commonly used drugs in TA patients, but there was no significant difference between two groups (Table 1).

Compared with those in inactive group, complements including C3 [1.00 (0.91–1.15) vs 1.19 (1.03–1.56), *P* < 0.001], C4 [0.22 (0.19–0.27) vs 0.25 (0.20–0.30), *P* = 0.040], and hemolytic complement (CH50, 59.3 ± 17.9 vs 66.4 ± 18.1, *P* = 0.015) were significantly higher in the active group. Moreover, serum levels of ESR, CRP, interleukin-6 (IL-6), platelets (PLT), globulin, immunoglobin A (IgA), and immunoglobin G (IgG) were also significantly higher in the active group compared with that in the inactive group (*P* < 0.05) (Table 1).

### The relationships between complements and other biomarkers with disease activity

Then, logistic analysis was performed to clarify the relationships between different biomarkers with disease activity. In univariate regression analysis, it revealed that C3 and CH50, together with PLT, globulin, CRP, IgA, IgG, and IL-6, were positively correlated with disease activity (OR > 1, *P* < 0.05). However, in multivariate regression analysis, C3 levels [odds ratio [OR] (95%CI) 10.710 (1.825–62.835), *P* = 0.009] and CRP [OR (95%CI) 1.041 (1.009–1.073), *P* = 0.011] were independently associated with active disease (Supplementary Table S1).

**Fig. 1:**
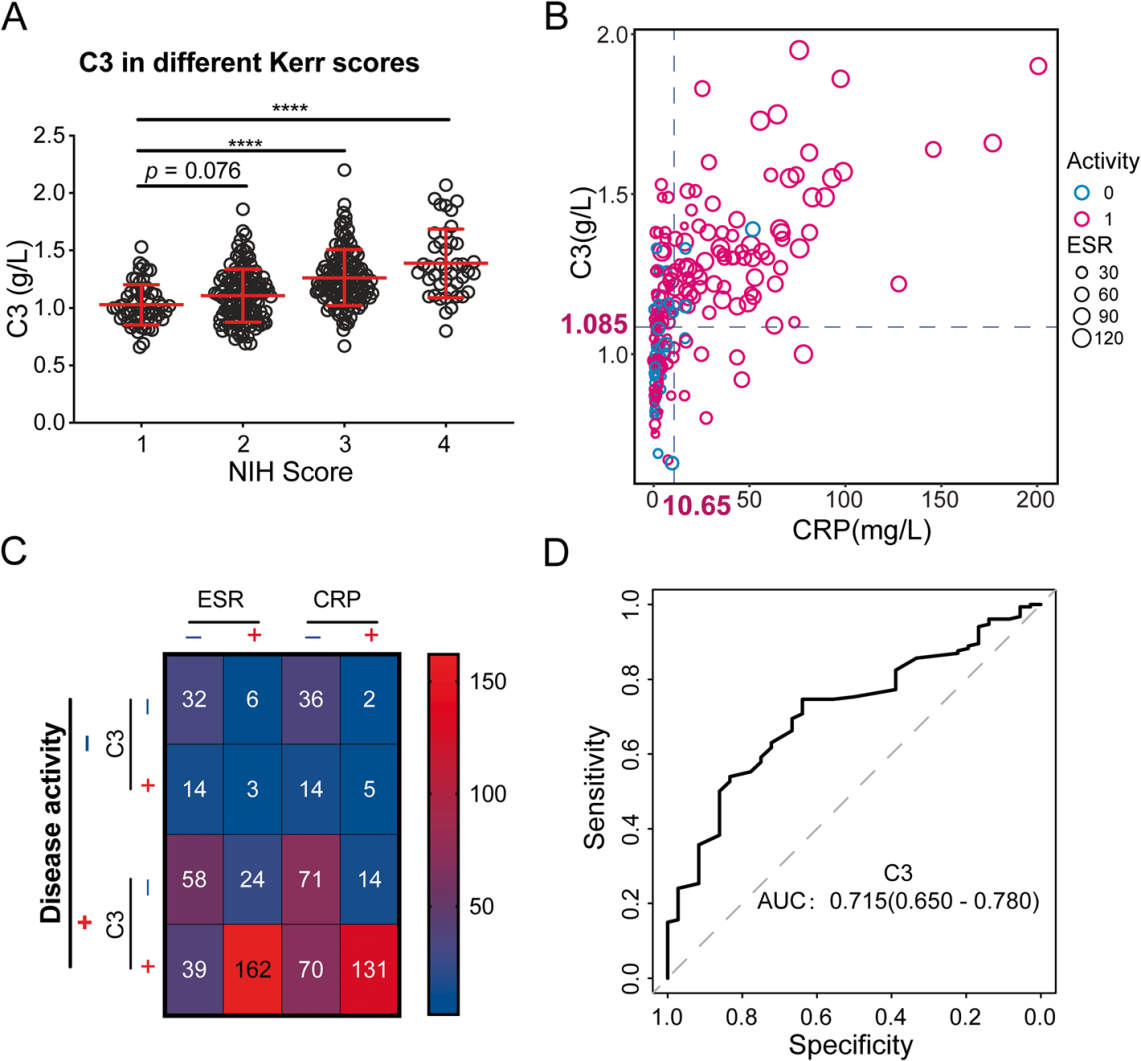
The value of C3 in identifying TA disease activity. **a** The distribution of C3 in different levels of Kerr score. **b** The scatter plot of C3, ESR, CRP, and disease activity (blue line—the cut-off reference line of C3 and CRP; dot size—ESR; dot colour—disease status, “red” indicates active disease status, “green” indicates inactive disease status). **c** The summary of C3, CRP, ESR, and disease activity in different subgroups based on the cut-off value. **d** The ROC curve of C3 to distinguish the disease activity

**Fig. 2:**
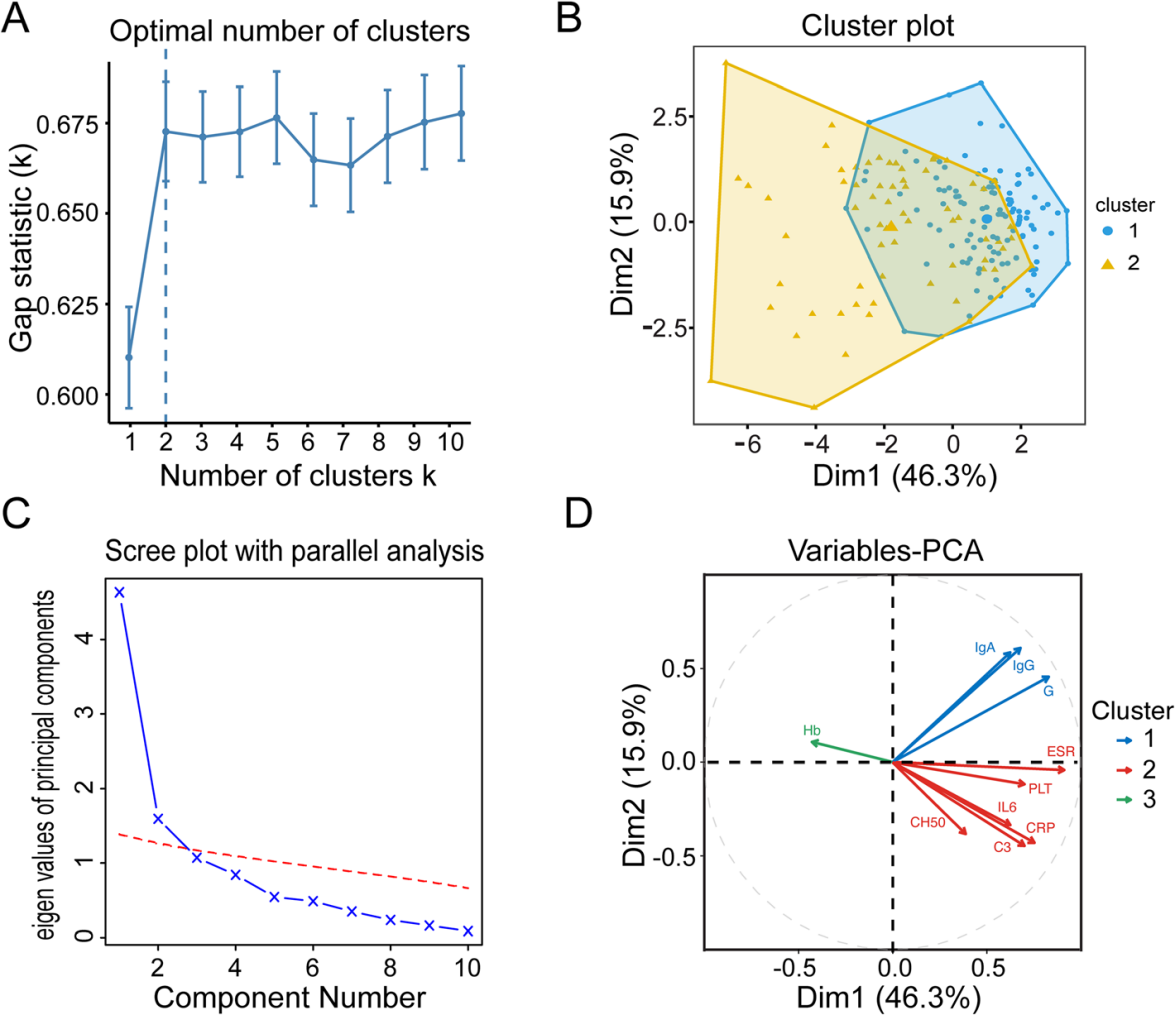
The cluster analysis and principal component analysis for variables associated with disease activity in TA. **a** Scree plot to explore the optimal number of the clusters. **b** Cluster analysis of the patients. **c** Scree plot to explore the optimal groups of the principal component analysis. **d** Principal component analysis based on the variables

In the scatter plot, C3, C4, and CH50 increased with Kerr score significantly and C3 was significantly positively associated with ESR, CRP, and IL-6 (*r* > 0.4, *P* < 0.001) (Fig. 1, Supplementary Fig. S2–3). Furthermore, cluster analysis showed that patients could be clustered into two groups based on C3, CH50, PLT, globulin, CRP, IgA, IgG, IL-6, ESR, and Hb levels. The principal component analysis revealed that C3, CH50, CRP, IL-6, PLT, and ESR were assigned to the same major component consisting of inflammatory biomarkers (Fig. 2). These data indicated that C3 levels were associated with the disease activity.

### The value of C3 and other biomarkers in identifying disease activity for TA

In the diagnosis test, the C3 cut-off value for identifying active disease was 1.085 g/L, with the sensitivity of 69.9%, the specificity of 66.7%, and the AUC of 0.715 (0.650–0.781). In comparison, the cut-off of CRP was 10.65 mg/L, with sensitivity of 50.7%, specificity of 82.4%, and the AUC of 0.703 (0.647–0.760). Moreover, the cut-off of ESR was 26.5 mm/H, with 63.8% sensitivity, 73.1% specificity, and the AUC of 0.766 (0.698–0.800). Further combined tests showed that combining CRP and C3 in parallel test, the sensitivity could be improved to 85.1%and the specificity was 55.0%. In serial test, the specificity was improved to 94.1%, with a sensitivity of 35.4% (Table 2, Fig. 1).

**Table 2:** The sensitivity and specificity of different biomarkers to distinguish the TA disease activity

Marker	AUC	Cut-off value	Sen	Spe	PLR	NLR	PPV	NPV
ESR*	0.766 (0.698–0.800)	26.5	0.638	0.731	2.371	0.495	0.923	0.780
C3	0.715(0.650–0.781)	1.085	0.699	0.667	2.099	0.451	0.911	0.667
CRP	0.703(0.647–0.760)	10.65	0.507	0.824	2.881	0.598	0.923	0.824
Globin	0.658(0.600–0.716)	29.05	0.543	0.736	2.057	0.621	0.890	0.736
Albumin^#^	0.623(0.556–0.690)	40.25	0.667	0.581	1.592	0.573	0.721	0.345
Haemoglobin^#^	0.620(0.559–0.681)	120.95	0.642	0.583	1.540	0.614	0.723	0.358
IgG	0.617(0.549–0.685)	13.32	0.503	0.736	1.905	0.675	0.895	0.736
CH50	0.614(0.529–0.699)	60	0.663	0.574	1.556	0.587	0.873	0.574
IL-6	0.610(0.533–0.686)	8.85	0.38	0.817	2.077	0.759	0.776	0.817
IgA	0.592(0.524–0.661)	2.865	0.438	0.743	1.704	0.756	0.881	0.743
PLT	0.566(0.506–0.627)	274.5	0.504	0.663	1.496	0.748	0.860	0.663
**Parallel test**								
ESR + C3	0.683(0.600–0.767)		0.891	0.488	1.740	0.223	0.884	0.615
CRP + C3	0.703(0.623–0.782)		0.851	0.550	1.891	0.271	0.895	0.670
ESR + CRP	0.713(0.638–0.788)		0.822	0.602	2.066	0.296	0.901	0.710
**Serial test**								
ESR + C3	0.758(0.700–0.816)		0.446	0.910	4.956	0.609	0.982	0.964
CRP + C3	0.703(0.639–0.766)		0.354	0.941	6.000	0.687	0.963	0.912
ESR + CRP	0.699(0.631–0.738)		0.323	0.953	6.832	0.710	0.960	0.918

*IgG* immunoglobin G, *CH50* median hemolytic complement, *IgA* immunoglobin A, *PLT* platelet, *PLR* positive likelihood ratio, *NLR* negative likelihood ratio, *PPV* positive predication value, *NPV* negative prediction value, *Sen* sensitivity, *Spe* specificity

^*^ESR belongs to the Kerr criteria and is used as the control here

^#^In the ROC curves, the disease activity status discrimination for albumin and haemoglobin is “0” (i.e. Inactive disease)

In addition, results of the NRI [OR (95%CI) 0.328 (0.224–0.431), *P* < 0.001] and IDI [OR (95%CI) 0.389 (0.312–0.466), *P* < 0.001] revealed that the introduction of C3 improved the ability of CRP to distinguish disease activity significantly.

### The validation of the value of combination of CRP and C3 in distinguishing active disease

In the internal validation, the 10-fold cross-validation revealed that the accuracy of the training group was more than 75% in the parallel test of CRP and C3, among which the maximal accuracy was 90.9% in the training group with the corresponding accuracy of 83.7% in the test group (Supplementary Table S2).

**Fig. 3:**
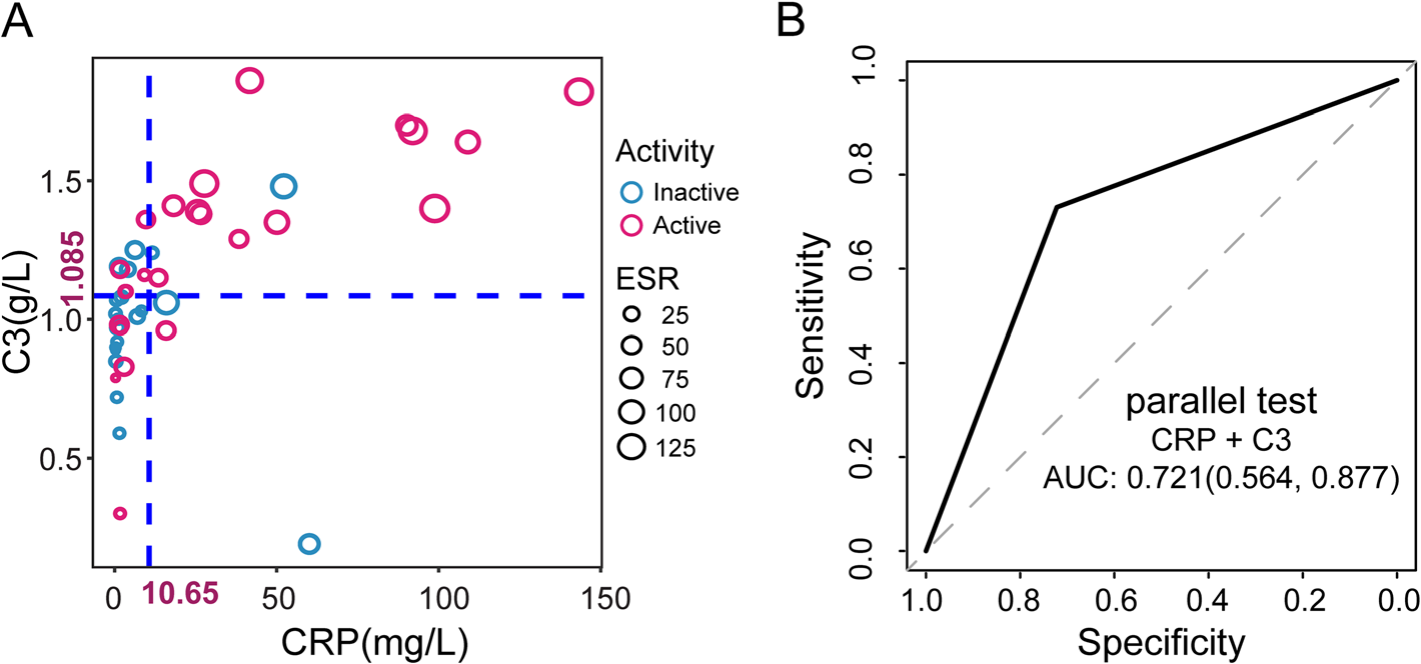
The external validation of CRP and C3 to identify the active disease based on the validating data in TA. **a** The distribution of C3, CRP, ESR, and disease activity in validation data (blue line—the cut-off reference line of C3 and CRP in original data; dot colour—disease activity status, “red” indicates active disease status, “green” indicates inactive disease status; dot size—ESR). **b** The ROC curve of parallel test (CRP and C3) to identify disease activity in validation data of TA

In the external validation, 53 cases of independent TA patients were employed to further validate the universality of the results, among whom, 24 (55.8%) cases were in active status according to the Kerr criteria. There was no significant difference between patients from the original dataset and validation dataset in age, sex, and levels of ESR, CRP, and C3. The AUC of CRP and C3 was 0.721 and 0.692, with the accuracy of 72.0% and 67.3%, respectively in validation dataset. The AUC was 0.721 with the accuracy of 72.7% in the parallel test of CRP and C3, while the AUC of serial test of CRP and C3 was 0.721 with the accuracy of 70.4%. The scatter plot also showed that the introduction of C3 could reclassify some patients with CRP below the cut-off value as active disease (Fig. 3, Supplementary Table S3–4).

## Discussion

To our knowledge, this is the first study to report the value of complement 3 in evaluating disease activity in TA. Our results revealed that elevated C3 had high value in identifying active disease for TA. In addition, the combination of C3 and traditional biomarker CRP could significantly improve the sensitivity or specificity to distinguish the active TA. One big advantage of the present study was that a validation analysis was performed and further confirmed the value of C3 for disease activity assessment, though minor differences were observed between original dataset and validation dataset, due to the limited validating sample size.

The convenient and feasible laboratory markers have been explored to evaluate TA disease activity for years, considering the complexity of the imaging examinations [16]. We found that the accuracy of C3 was similar to ESR, but higher than CRP in the present study. Moreover, the sensitivity of C3 was much better than that of ESR and CRP, which could be validated by an independent group of patients. These data strongly supported that C3 might become a new valuable biomarker for evaluating disease activity in TA.

Inflammation is the foundation of vascular fibrosis and remodelling in TA; thus, assessing disease activity precisely is very important to prevent disease evolution [2, 3]. We found that C3 and inflammatory indices including CRP, IL-6, ESR, and PLT belonged to the same component and were positively correlated with active disease and could classify patients into two groups. Moreover, C3 was correlated with CRP, ESR, and IL-6 levels, indicating the role of C3 in evaluating inflammation and identifying disease activity [16]. According to previous reports, IL-6 was involved in the pathogenesis of TA and has become an important intervention target recent years [17, 18]. However, in our present study, IL-6 showed a relatively lower AUC in identifying active disease, compared with C3.

To improve evaluating disease activity of TA, we performed the parallel and serial tests. The results indicated that the combination of CRP and C3 could significantly improve the sensitivity or specificity to identify active disease in TA. Further validation also confirmed the accuracy of the model in the present study. Considering the multiple components of conventional methods to identify disease activity such as Kerr criteria [19, 20], a single biomarker was difficult to distinguish the active status of the disease for the moment, but combined tests could compensate the shortcomings according to the study. However, which combination was the best strategy was still needed to be further investigated and validated in other cohorts.

C3 might be also involved in the pathogenesis of TA. In TA, the elevated autoantibody anti-epithelial cell antibodies (AECA) serum could mediate the complement-dependent cytotoxicity, leading to the vascular pathogenic lesions [21, 22]. Moreover, infectious antigens might boost the levels of MHC I chain-related A (MICA), initiating acute inflammation [23]. In this process, proteins such as CRP, targeting microbes, could bind to phosphocholine of the cell membrane, activating C3 through the classical pathway, promoting macrophage elimination of antigens such as debris in active TA [5], resulting in the increased inflammation in the vascular lesions. Accordingly, the upstream protein of C3 and C4b were much higher in the serum of TA as well [16]. These phenomena were also consistent with the finding that C3 was associated with the active disease. Further mechanisms and causal relations between C3 and other inflammatory index and disease activity should be explored in the future.

There were limitations in the present study. First, the study was based on the ECTA cohort and the results needed to be further validated in other TA cohorts in the future. Second, we found that C3 was valuable to distinguish active disease, compared with C4 and CH50, but whether other complement components such as inflammatory C3a and C5a could reflect the active disease remained unclear [12].

## Conclusions

In conclusion, C3 is a potential biomarker for disease activity evaluation in TA, which could also improve the diagnosis ability of other markers.

## Supplementary Information

**Additional file 1: Supplementary Table S1.** The logistic regression analysis of TA disease activity. **Supplementary Table S2**. The 10-fold cross-validation to evaluate the accuracy of the parallel test CRP and C3 with the original dataset in the internal validation. **Supplementary Table S3**. The characteristics of patients in the independent external validation dataset. **Supplementary Table S4**. The detailed diagnosis result of the external validation. **Supplementary Figure S1.** The flowchart of the study. **Supplementary Figure S2**. The distribution of C4 and CH50 in different level of Kerr score. **Supplementary Figure S3**. The correlation analysis of variables.

## Data Availability

The datasets used and/or analysed during the current study are available from the corresponding author on reasonable request.

## Abbreviations

TA: Takayasu arteritis
ESR: Erythrocyte sedimentation rate
CRP: C-reactive protein
C3: Serum complement 3
MMP: Matrix metalloproteinases
PTX-3: Pentraxin-3
SLE: Systemic lupus erythematosus
AAV: ANCA-associated vasculitis
NRI: Net reclassification index
IDI: Integrated discrimination index
MRA: Magnetic resonance angiography
ROC: Receiver operating characteristic
IL-6: Interleukin-6
PLT: Platelets
IgA: Immunoglobin A
IgG: Immunoglobin G
OR: Odds ratio
AECA: Anti-epithelial cell antibodies
MICA: MHC I chain-related A
