# Supplementary material for: Serum complement 3 is a potential biomarker for assessing disease activity in Takayasu arteritis
Source: Arthritis Res Ther. 2021 Feb 24;23:63. doi: 10.1186/s13075-021-02433-x (PMC7903686; doi:10.1186/s13075-021-02433-x)
Supplement: Supplementary file 1 — Additional file 1: Supplementary Table S1. The logistic regression analysis of TA disease activity. Supplementary Table S2. The 10-fold cross-validation to evaluate the accuracy of the parallel test CRP and C3 with the original dataset in the internal validation. Supplementary Table S3. The characteristics of patients in the independent external validation dataset. Supplementary Table S4. The detailed diagnosis result of the external validation. Supplementary Figure S1. The flowchart of the study. Supplementary Figure S2. The distribution of C4 and CH50 in different level of Kerr score. Supplementary Figure S3. The correlation analysis of variables. [file 13075_2021_2433_MOESM1_ESM.docx]

**Supplementary Materials**

**SUPPLEMENTARY TABLES**

**Supplementary Table S1.** The logistic regression analysis of TA disease activity.

|  | **Univariate analysis** | |  | **Multivariate analysis** | |
| --- | --- | --- | --- | --- | --- |
|  | **OR (95%CI)** | ***P* value** |  | **OR (95%CI)** | ***P* value** |
| Age, years | 1.005(0.990 – 1.020) | 0.540 |  |  |  |
| Sex | 0.746(0.443 – 1.256) | 0.270 |  |  |  |
| Course, months | 1.001(0.999 – 1.004) | 0.386 |  |  |  |
| Hemoglobin, g/L | 0.981(0.969 – 0.992) | < 0.001^*^ |  |  |  |
| Platelet, ×10^9^/L | 1.002(1.000 – 1.005) | 0.045^*^ |  |  |  |
| Albumin, g/L | 1.001(0.997 – 1.004) | 0.718 |  |  |  |
| Globin, g/L | 1.086(1.042 – 1.131) | < 0.001^*^ |  |  |  |
| CRP, mg/L | 1.036(1.019 – 1.054) | < 0.001^*^ |  | 1.041(1.009 – 1.073) | 0.011^*^ |
| IL-6, pg/mL | 1.048(1.004 – 1.094) | 0.031^*^ |  |  |  |
| IgG, g/L | 1.099(1.030 – 1.172) | 0.004^*^ |  |  |  |
| IgA, g/L | 1.297(1.025 – 1.600) | 0.015^*^ |  |  |  |
| C3, g/L | 35.366(8.311 – 150.137) | < 0.001^*^ |  | 10.710(1.825 – 62.835) | 0.009^*^ |
| C4, g/L | 1.005(0.980 – 1.030) | 0.709 |  |  |  |
| CH50, g/L | 1.021(1.004 – 1.039) | 0.017^*^ |  |  |  |

**Notes**.

a. Abbreviations: CRP, C-reactive protein; IL-6, interleukin-6; IgA, immunoglobin A; IgG, immunoglobin G; C3, complement 3; C4, complement 4; CH50, median hemolytic complement.

b. Method: The univariate logistic regression analysis was performed with disease activity as the dependent variable.

c. ^*^ *P* < 0.05.

**Supplementary Table S2**. The 10-fold cross-validation to evaluate the accuracy of the parallel test CRP and C3 with the original dataset in the internal validation.

| Group ID | Accuracy of training group | Accuracy of test group |
| --- | --- | --- |
| 1 | 0.8235294 | 0.8428094 |
| 2 | 0.9090909 | 0.8366667 |
| 3 | 0.8181818 | 0.8433333 |
| 4 | 0.7647059 | 0.8494983 |
| 5 | 0.8181818 | 0.8433333 |
| 6 | 0.8484848 | 0.84 |
| 7 | 0.9090909 | 0.8333333 |
| 8 | 0.8787879 | 0.8366667 |
| 9 | 0.8484848 | 0.84 |
| 10 | 0.7941176 | 0.8461538 |

**Notes**.

a. The best accuracy of test group is 0.91, and the corresponding accuracy of the training group is 0.84.

b. ‘logit’ method was used in the analysis.

c. The patients in original dataset were randomly divided into training group and test group with the ratio of 7:3.

**Supplementary Table S3**. The characteristics of patients in the independent external validation dataset.

|  | Original dataset | Validation dataset | *P* value |
| --- | --- | --- | --- |
|  | (n=519) | (n=53) |  |
| Age, years | 32(24 – 45) | 31(25 – 48.5) | 0.718 |
| Sex, n (%) | 427(82.3) | 45(84.9) | 0.708 |
| Hemoglobin, g/L | 119(106 – 130) | 114(101 – 122) | 0.031^*^ |
| WBC, ×10^9^/L | 7.46(5.96 – 9.76) | 8.04(5.99 – 9.33) | 0.885 |
| Platelet, ×10^9^/L | 265(213 – 342) | 287(220 – 350) | 0.564 |
| ESR, mm/H | 33(14 – 60) | 31(9 – 55) | 0.267 |
| CRP, mg/L | 7.5(1.9 – 30.8) | 7.7(1.5 – 26.9) | 0.624 |
| IL-6, pg/ml | 5.3(2.5 – 11.2) | 6.6(3.4 – 14.7) | 0.207 |
| C3, g/L | 1.15(0.99 – 1.33) | 1.10(0.97 – 1.33) | 0.520 |
| C4, g/L | 0.24(0.20 – 0.29) | 0.23(0.19 – 0.27) | 0.176 |
| CH50, g/L | 65.1(53.9 – 78.3) | 68.8(55.2 – 80.7) | 0.463 |

**Notes**. a. Abbreviations: WBC, white blood cells; ESR: erythrocyte sedimentation rate; CRP: C-reactive protein; IL-6: interleukin-6; C3: complement 3; C4: complement 4; CH50: median hemolytic complement.

b. ^*^*P* < 0.05.

**Supplementary Table S4**. The detailed diagnosis result of the external validation.

| **Marker** | **AUC** | | | | **Cut-off value** | | **Sen** | **Spe** | **PLR** | **NLR** | **PPV** | **NPV** | **Accuracy** |
| --- | --- | --- | --- | --- | --- | --- | --- | --- | --- | --- | --- | --- | --- |
| C3 | 0.721(0.575 – 0.867) | | | | 1.085 | | 0.714 | 0.727 | 2.615 | 0.393 | 0.769 | 0.667 | 0.720 |
| CRP | 0.692(0.533 – 0.850) | | | | 10.65 | | 0.600 | 0.762 | 2.521 | 0.525 | 0.750 | 0.615 | 0.673 |
| **Parallel test** | |  |  |  | |  |  |  |  |  |  |  |  |
| CRP + C3 | 0.721(0.564 – 0.877) | | | |  | | 0.792 | 0.650 | 2.263 | 0.320 | 0.731 | 0.722 | 0.727 |
| **Serial test** |  | | | |  | |  |  |  |  |  |  |  |
| CRP + C3 | 0.721(0.568 – 0.874) | | | |  | | 0.542 | 0.900 | 8.621 | 0.531 | 0.867 | 0.622 | 0.704 |

**Notes**. a. Abbreviation: AUC, area under the curve; C3, complement 3; CRP, C-reactive protein; Sen, sensitivity; Spe, specificity; PLR, positive likelihood ratio; NLR, negative likelihood ratio; PPV, positive prediction value; NPV, negative prediction value.

**SUPPLEMENTARY FIGURES.**

**Supplementary Figure S1.** The flowchart of the study.


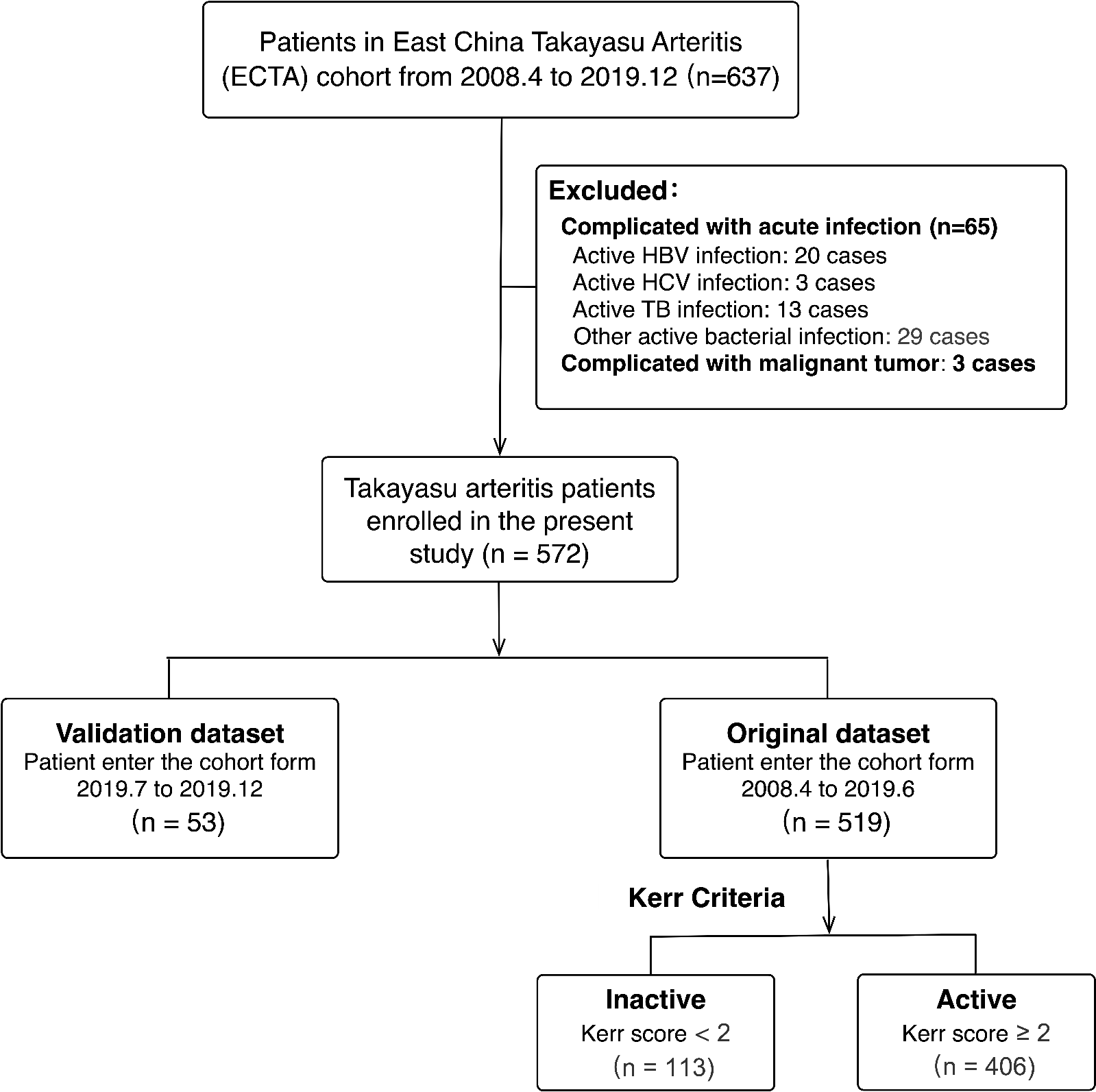


**Notes.**

a. HBV, hepatitis B virus; HCV, hepatitis C virus; TB: tuberculosis; Other active bacterial infection includes pneumonia, urinary tract infection, infective endocarditis, and syphilis infection.

**Supplementary Figure S2**. The distribution of C4 and CH50 in different level of Kerr score.


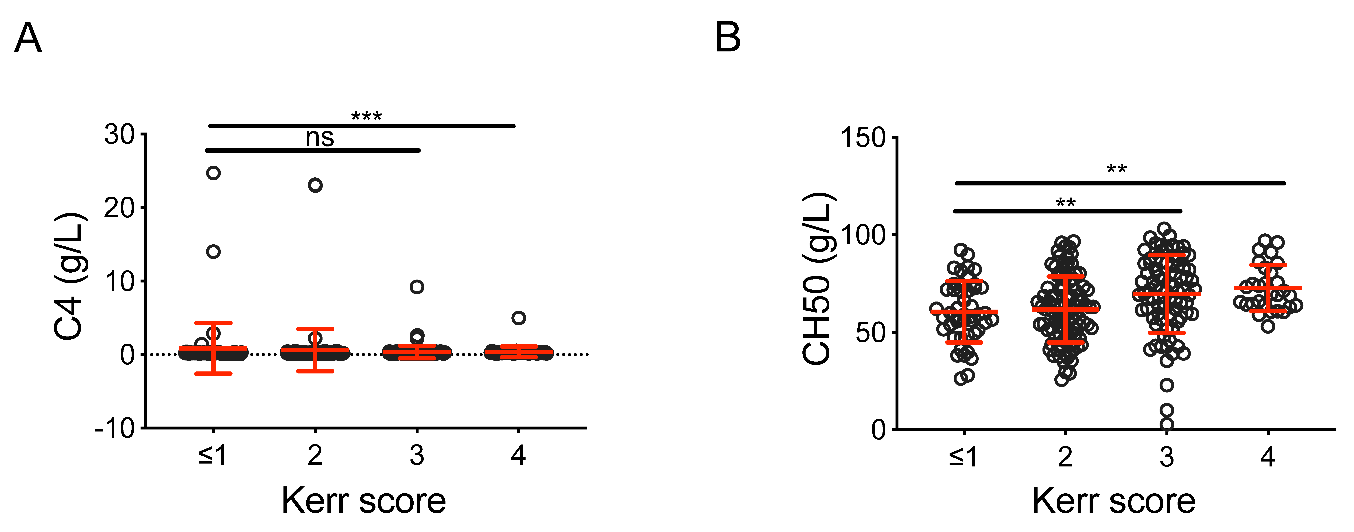


**Notes.**

a. Abbreviations: C4, complement 4; CH50, median hemolytic complement; ns., non-significant.

b. ^**^ *P* < 0.01; ^***^ *P* < 0.001.

**Supplementary Figure S3**. The correlation analysis of variables.

1. The correlation analysis of C3 and ESR;
2. The correlation analysis of C3 and CRP;
3. The correlation analysis of C3 and IL-6;


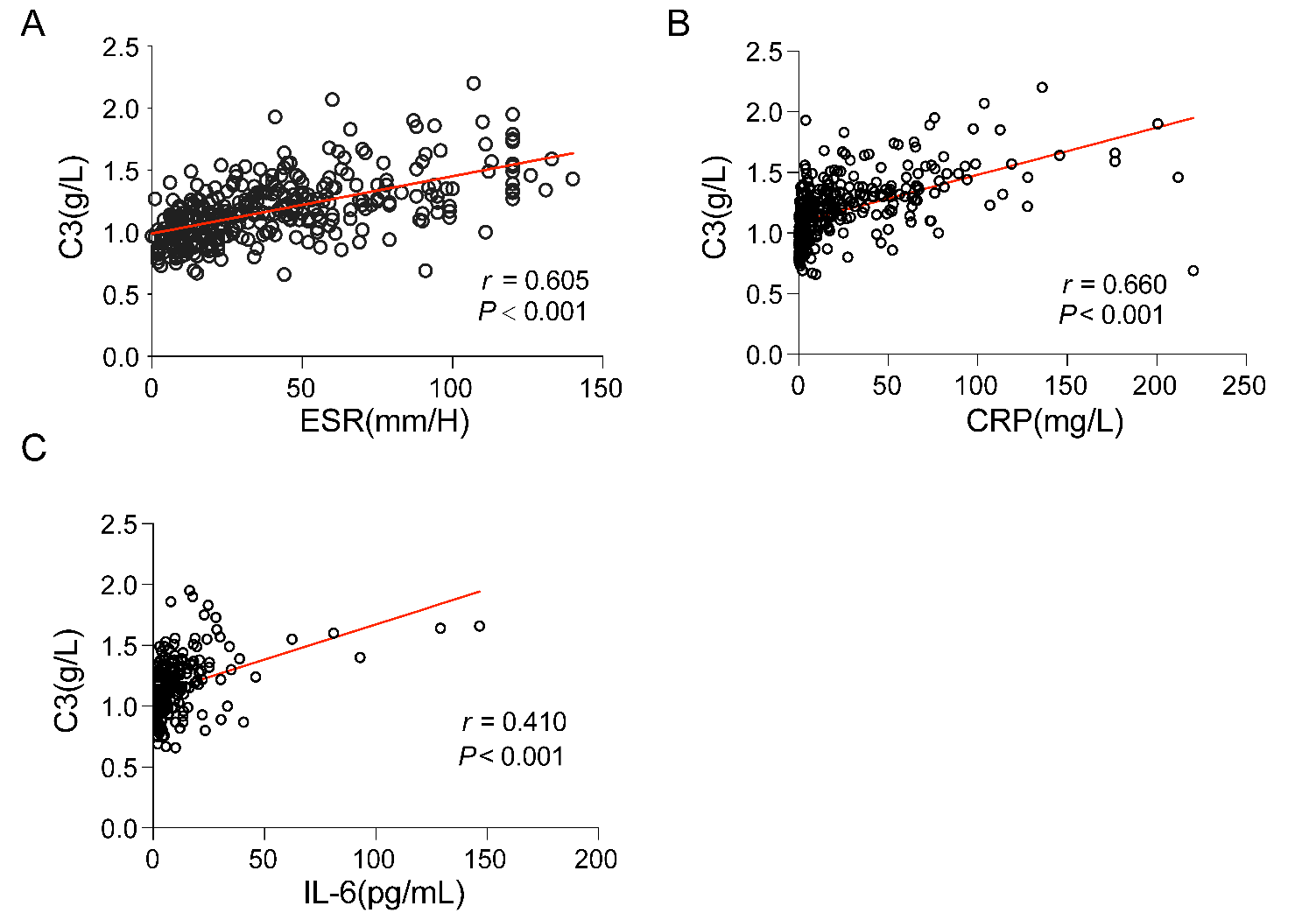


**Notes.**

a. Methods: The spearman correlation analysis was employed to observe the relationship between C3 and IL-6, ESR, and CRP.

b. Abbreviations: C3, complement 3; ESR, erythrocyte sedimentation rate; IL-6, interleukin-6; CRP, C-reactive protein.

c. ^*^*P* < 0.05.
